# A Call for Responsible Estimation of Global Health

**DOI:** 10.1371/journal.pmed.1001003

**Published:** 2010-11-30

**Authors:** Wendy J. Graham, Sam Adjei

**Affiliations:** 1 Professor of Obstetric Epidemiology, School of Medicine and Dentistry, University of Aberdeen, United Kingdom; 2 Centre for Health and Social Services (CHeSS), Accra, Ghana

**Summary Points**

- There is new urgency and potential to deliver leadership on the process of global estimation of women's and children's health.
- Recent controversies over global estimates highlight fundamental questions about the performance of the “suppliers” of the figures and the needs of the “clients”.
- Stakeholders in the process are now even more diverse, and include country governments, bilateral and multi-lateral agencies, academics, professional associations, and non-governmental organisations, and newer members from global philanthropic organisations and the business community.
- We propose responsible estimation of global health, which is stakeholder-centric, accountable, and transparent, and which has a clear leader.

*This article is part of a cluster of five articles on global health estimates.*

On September 22, 2010, a new Global Strategy for Women's and Children's Health was launched by the United Nations Secretary-General and over US$40 billion in resources pledged [1]. Two key drivers of this call to action are the short period remaining to achieve the UN's Millennium Development Goals (MDGs) and the realisation that the goal for maternal health is off target. The Global Strategy argues that the world has failed to invest enough in the health of women and children, presses for more intensified effort, and articulates the projected “gain” in terms of saving 16 million lives by 2015. Here we seek to highlight the potential of this latest initiative to deliver leadership on the process of global estimation of women's and children's health.

At the heart of recent debates and controversies over global estimates—be these on immunisation [2], child deaths [3], or HIV/AIDS [4]—lie fundamental questions about the performance of the “suppliers” of the figures and the needs of the “clients”. Let us illuminate the situation using maternal mortality. Until very recently, the most up-to-date estimates of maternal mortality at world, region, and national levels were for 2005, developed by an inter-agency group comprising the World Health Organization, the United Nations Children's Fund, the United Nations Population Fund, and the World Bank. We refer to this as a global estimation exercise since it uses a standard approach to handling national data to enable comparisons over time and between geographic areas. Whilst aimed essentially at creating a global picture, the modelled estimates provide the only national figures on maternal mortality for some low-income countries. Since September 2010, however, two different sets of estimates and trends are available—one produced by academics at the Institute for Health Metrics and Evaluation (IHME) at the University of Washington and the other by the UN Inter-Agency group [5],[6].

Why does this matter? Reactions from the “clients” for the estimates are as diverse as the group itself. These stakeholders include long-standing users of this information—country governments, bilateral and multi-lateral agencies, academics, professional associations, and non-governmental organisations—as well as newer users from global philanthropic organisations and the business community. However, for all of them, it is not hard to imagine the potential for confusion from there being two sets of estimates for such a key MDG target. This is perhaps felt most acutely at the country level, where earlier remarks and requests for clarity about the IHME estimates from individuals and groups based in low- and middle-income countries [7],[8] are now likely to be superseded by other questions: why are the estimates different and which set should we rely upon? At a global and regional level, and indeed for some countries, the IHME and UN estimates of maternal mortality are thankfully and not surprisingly similar. However, there are other countries, particularly in sub-Saharan Africa, where the difference is significant and unhelpful to important and sensitive decisions on progress and resource allocation (see Figure 1). Some countries will thus opt to use their existing and nationally “owned” estimates, and others will embrace one or the other of the globally produced figures.

**Figure 1:**
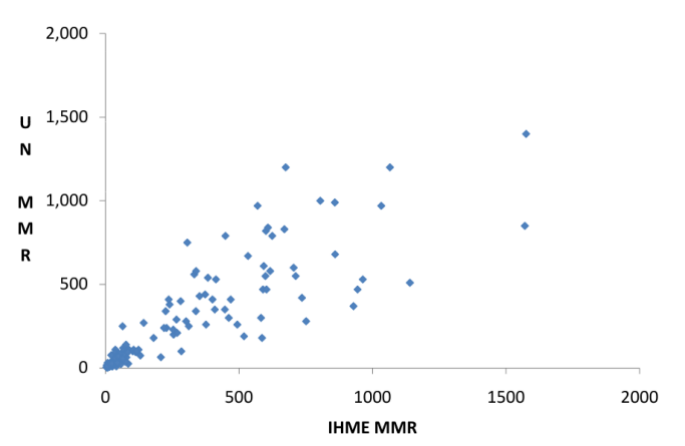
Comparison of two sets of estimates for the maternal mortality ratio for 171 countries, 2008. Estimates are from the UN inter-agency group (UN) [6] and the IHME [5]; maternal mortality ratio (MMR) is maternal deaths per 100,000 live births. Those countries with very similar estimates from the two sources lie essentially along the 45-degree line in this chart. Countries lying above such a line have estimates from the UN higher than those from IHME, and vice versa for countries below the line. For example, one country has an MMR estimate of 1,200 from the UN versus 675 from the IHME.

So what's to be done? The need to reduce the reliance on modelled estimates is well accepted—a reliance that largely reflects the poor state of investment in health information systems in low-income countries. However, this will not remove the requirement for global comparative processes, and so efforts to improve current practice are still warranted. This brings us back to the potential of the Global Strategy, with its promising narrative around accountability: national ownership of results, strengthening countries' capacity, and harmonising mechanisms for tracking progress. Encouraging signs also lie in the frequent mention of leadership in the main document and in the press release: “Today we are witnessing the kind of leadership we have long needed” [9].

But perhaps the most reassuring words are found in the simple phrase “we all have a role to play” [1]. This mantra is, in our view, entirely correct, much like the idea that all instruments and musicians play a role in an orchestra. The analogy [10] is useful because it highlights both the vacant position of a conductor among the current community of estimate suppliers and the comparative neglect of the audience or clients. The global pool of expertise available to grapple with complex modelling methods is comparatively small, and although diversity among the approaches and contributors is to be encouraged, eventually there must be some alignment for the sake of the end users. How to achieve this requires the mastery of a conductor—a visionary who sees global estimation as a process continuing well after the figures are released in order to satisfy the audience. The analogy should be used cautiously here, since conducting various groups producing robust global estimates, be this for maternal mortality or any other health parameter, does not necessarily imply an individual. The “conductor” could be an organisation, though recent unsuccessful efforts to bring together the two groups generating maternal mortality estimates perhaps hints at the need for a maestro [11].

Respecting the audience whilst also maintaining balance in the orchestra is indeed the role of the conductor. The best estimates will ultimately come from the creative power of many players, and not by allowing only one approach or group to dominate at the expense of others. What must happen eventually, however, is that the players harmonise for the sake of the audience. This is what we refer to as responsible estimation (Box 1). The Global Strategy for Women's and Children's Health is an opportunity to appoint a conductor and satisfy the audience. We look forward eagerly to its opening performance.

**Box 1. Responsible Estimation of Global Health**

A co-ordinated estimation process that is stakeholder-centric and has the requisite leadership to harness and harmonise inputs from all relevant technical players. The process must engage with stakeholders from the outset, continue to work transparently and consultatively with them during the creation of new figures, and support and build capacity in country to use, own, and improve the estimates.

## Abbreviations

IHME: Institute for Health Metrics and Evaluation
MDG: Millennium Development Goal
